# Microstructural and Rheological Properties of Camel and Bovine Milk Fermented with Five Lactic Acid Bacteria Strains

**DOI:** 10.3390/foods15030546

**Published:** 2026-02-04

**Authors:** Sifatun Nesa Ali, Syed Muhammad Asim, Nadiya Samad, Mutamed Ayyash, Afaf Kamal-Eldin

**Affiliations:** 1 Department of Food Science, College of Agriculture and Veterinary Medicine, United Arab Emirates University, Al Ain P.O. Box 15551, United Arab Emiratesmutamed.ayyash@uaeu.ac.ae (M.A.); 2 Big Data Center, United Arab Emirates University, Al Ain P.O. Box 15551, United Arab Emirates

**Keywords:** lactic acid bacteria, fermentation, camel milk, bovine milk, microscopy, rheology

## Abstract

This study assessed the fermentation performance of five lactic acid bacteria (*Streptococcus thermophilus*, *Lactobacillus delbrueckii* ssp. *bulgaricus*, *Lb. helveticus*, *Lb. casei*, and *Lactiplantibacillus plantarum*) in camel milk (CM) and bovine milk (BM) at 42 °C for 48 h. Fluorescence microscopy revealed lower bacterial viability in fermented CM compared to BM. Acidification kinetics varied significantly between CM and BM, and proteolysis was more pronounced in fermented CM (*p* < 0.001), with OPA concentrations ~1.3–1.5-fold greater in CM across all strains during fermentation. Scanning electron microscopy revealed more porous, loose protein matrices in fermented CM than in BM, in line with the rheological analyses showing weaker gel networks and lower rheological strength in fermented CM. *Lb. casei* demonstrated superior adaptability, enhanced viability, balanced acidification, and favorable rheological properties in both milks, highlighting its potential as a possible starter or adjunct culture in fermented dairy products.

## 1. Introduction

Camel milk (CM) is increasingly produced and consumed in arid and semiarid regions worldwide [1]. Recently, it has gained popularity owing to its distinct nutritional and therapeutic properties, particularly antidiabetic effects [2,3]. Notably, CM contains elevated levels of protective proteins, including immunoglobulins, lactoferrins, lysozymes, and lactoperoxidases [4], as well as peptides [2,5], which may contribute to its antimicrobial activity and longer shelf life [6]. Yogurt fermentation bacteria grow more slowly in CM than in bovine milk (BM), likely due to the combined effects of these components [7].

The characteristics of yogurt gels, particularly microstructure, gel strength, rheology, and syneresis, are markedly influenced by milk type and fermentation process [8]. Beyond slow fermentation rates, CM exhibits weak coagulation capacity, which results in fragile gels and watery mouthfeel [7], complicating its processing into traditional fermented dairy products, such as yogurt and cheese [9,10]. A key difference between CM and BM is the absence of β-lactoglobulin, low levels of κ-casein, and the presence of large casein micelles in CM [11,12,13]. The formation of β-lactoglobulin–κ-casein complexes during heat treatment before fermentation is crucial for the formation of set yogurt from BM [14].

Traditional dairy starter cultures include *Streptococcus thermophilus* and *Lactobacillus delbrueckii* ssp. *bulgaricus*, which synergistically affect acidification and texture development [15,16]. *S. thermophilus* enables rapid acidification [17], whereas *Lb. bulgaricus* enhances proteolysis and flavor development [15,18]. Unlike the set yogurt produced from BM, these cultures fail to form hard curd in CM, creating a product with weak coagulation and a watery texture that is more suitable as drinkable yogurt [19]. Other bacteria may perform differently in CM and BM [20]. For example, *Lactobacillus helveticus* exhibits strong proteolytic activity with antibacterial effects [7,21], whereas *Lactobacillus casei* [22] and *Lactiplantibacillus plantarum* provide probiotic benefits and enhance the microbiological safety of fermented milk [23,24]. In addition, *Lb. casei* [25] and *L. plantarum* are associated with improved bacterial viability and increased viscosity [26]. Given these differences in metabolic activity and adaptability, this study evaluated the performance of five lactic acid bacteria, namely, *S. thermophilus, Lb. bulgaricus, Lb. helveticus, Lb. casei,* and *L. plantarum*, in CM fermentation. Their performance was compared with that in BM fermentation at 42 °C for 48 h, assessing bacterial adaptation, survival, acidification kinetics, proteolytic activity, microstructure, and rheological behavior.

## 2. Materials and Experiments

### 2.1. Materials and Chemicals

Man, Rogosa, and Sharpe (MRS) broth, peptone water, and plate count agar (LAB M, Lancashire, UK) were used as growth media. Sodium hydroxide, hydrochloric acid, *ortho*-phthalaldehyde (OPA), sodium dodecyl sulfate, sodium tetraborate, acetonitrile, β-mercaptoethanol, urea, methanol, and ethanol were purchased from Sigma-Aldrich (St. Louis, MO, USA). Laemmli buffer, polyacrylamide, tris-HCL, acrylamide, bis–tris solution, sodium dodecyl sulfate (SDS), N,N,N′,N′-tetramethylethylenediamine (TEMED), ammonium persulfate, and Coomassie dye were obtained from Bio-Rad Laboratories (Hercules, CA, USA).

### 2.2. Bacteria Cultivation

*S. thermophilus* DSM 20617, *Lb. delbrueckii* ssp. *bulgaricus* DSMZ 20081, *Lb. helveticus* DSM 20075, *Lb. casei* DSMZ 20207, and *L. plantarum* DSMZ 2648 were obtained from Leibniz-Institute DSMZ-Deutsche Sammlung von Mikroorganismen und Zellkulturen GmbH (Braunschweig, Germany). Each vial, comprising approximately 0.5 g of concentrated bacterial suspension, was resuspended in 0.5 mL of MRS broth containing 50% glycerol (1:1, *w*/*v*) and preserved at −80 °C. Prior to use, after thawing, 0.16 mL of suspension was transferred and activated into 3.33 mL of fresh MRS medium.

### 2.3. Milk Fermentation

Commercial skimmed CM and BM powders were procured from local stores in Al-Ain, UAE, to assure consistency and to emphasize protein–microbial interactions, as whole milk offered considerable variability in fat content. Reconstituted milk samples were prepared by dissolving skimmed milk powder in deionized water at 12% (*w*/*v*) and stored at 4 °C until use. Starter cultures were activated by inoculating 1% (*v*/*v*) of each bacterium into MRS broth and incubating anaerobically at 37 °C for 24 h. CM and BM samples were sterilized at 105 °C for 10 min, cooled to 42 °C (required fermentation temperature), and inoculated with 1% (*v*/*v*) of the activated starter cultures. Fermentation was performed at 42 °C for 48 h in triplicate [7]. Although not optimal for all bacteria, the same fermentation conditions were used to reach the target pH in camel (pH ~4.3) and bovine milk (pH ~4.6). Although 42 °C exceeds the optimal growth temperature for *L. plantarum* and *Lb. casei* (typically ~37 °C) [27,28], this temperature is commonly employed in studies involving thermophilic starter cultures [29,30].

### 2.4. Bacterial Growth Analysis

Bacterial populations in fermented CM and BM were determined following vortex homogenization. In brief, 1 mL of each fermented sample was serially diluted ten-fold in 0.1% sterile peptone water. Total bacterial counts were obtained in triplicate using the pour-plate technique with MRS medium. For population analysis, the diluted samples were plated on MRS agar in triplicate and incubated anaerobically at 37 °C for 48 h using an anaerobic jar system (Don Whitley Scientific Ltd., West Yorkshire, UK) [31].

### 2.5. Live and Dead Bacteria Analysis

Live and dead bacteria were quantified using the LIVE/DEAD^®^ BacLight™ Bacterial Viability and Counting Kit (Invitrogen, Molecular Probes, Carlsbad, CA, USA) containing a microsphere suspension and two nucleic acid dyes, SYTO 9 and propidium iodide (PI), which selectively stain live and dead bacteria, respectively. For viability analysis, 1 mL of each sample was mixed with 9 mL of phosphate-buffered saline and serially diluted to 10^2^ using the same buffer. A 1 mL aliquot from each dilution was mixed with 3 μL of 1:1 SYTO 9: PI dye mixture and vortexed at 500 rpm for 15 min in the dark at room temperature to ensure binding between the dyes and bacterial nucleic acids. A 5 μL stained sample was placed on a poly-L-lysine-coated slide (Sigma, Barcelona, Spain), covered, and sealed with Vaseline to prevent evaporation [32]. Bacterial enumeration was performed using a Motic epifluorescence microscope (BX20/0.50) (Motic, Richmond, BC, USA) equipped with FITC (Ex/Em = 488/530 nm) and TRITC (Ex/Em = 540/620 nm) channels. Green fluorescence indicated live cells, whereas red fluorescence indicated compromised or dead cells as per the manufacturer’s protocol. Live and dead bacteria viability was observed only for initial visualization rather than for strict quantitative comparison.

### 2.6. Titratable Acidity (TA) and pH Measurement

Titratable acidity was measured using 0.1 N Sodium hydroxide (NaOH) and presented as a percentage (TA%) of lactic acid, following ISO/IDF standards (2012) [33]. Before titration, fermented milk samples were equilibrated to ambient temperature. Subsequently, pH was determined with a calibrated Start-3100 digital pH meter (OHAUS Corporation, NJ, USA) [19].

### 2.7. Degree of Proteolysis

Water-soluble extracts were prepared by adjusting the fermented milk samples to pH 4.6, centrifuging at 9000× *g* and 4 °C for 15 min, and filtering through a 0.45 µm nylon filter. These extracts were stored at −20 °C until analysis. The degree of protein hydrolysis in the samples was evaluated using the OPA assay [34]. OPA reagent preparation involved dissolving 0.5 g of each sodium tetraborate and SDS, as well as 40 mg of OPA (dissolved in 1 mL of methanol), in 45 mL of deionized water. To this solution, 100 μL of β-mercaptoethanol was added, and the final volume was adjusted to 50 mL before vortexing for 1 min to ensure thorough mixing. Subsequently, each 100 μL water-soluble extract was mixed with 1 mL of freshly prepared OPA reagent for 5 s and incubated in the absence of light for 2 min, after which absorbance was recorded at 340 nm with a Jenway UV/VIS spectrophotometer (Model 6300, Cole-Parmer, Staffordshire, UK). Milli-Q water with OPA reagent served as the blank. All measurements were performed in triplicate, and means were reported to improve accuracy.

### 2.8. Sodium Dodecyl Sulfate–Polyacrylamide Gel Electrophoresis (SDS-PAGE)

SDS-PAGE was performed to analyze protein profiles in fermented CM and BM samples as described by [35]. In brief, each 0.2 g sample was vortexed using 4.17 mL of urea solution (8 M) for 1 min, incubated at 37 °C for 2 h, and centrifuged at 9150× *g* and 4 °C for 35 min. The resulting supernatant (10 μL) was added to 30 μL of freshly prepared 50 mM dithiothreitol in 4× Laemmli buffer, vortexed, and heated for 5 min at 90 °C. Subsequently, each prepared 6 μL sample was transferred onto a polyacrylamide gel composed of resolving gel (12%) and stacking gel (4%) layers. The 12% resolving gel was prepared with 3.75 mL of 1.5 M Tris-HCl buffer (pH 8.8), 6 mL of 30% acrylamide/Bis solution (29:1), 5.03 mL of deionized water, 150 μL of 10% SDS, 7.5 μL TEMED, and 75 μL ammonium persulfate (10% APS). The stacking gel (4%) comprised 3.78 mL Tris-HCl buffer (0.5 M, pH 6.8), 1.98 mL acrylamide/Bis solution (30%), 9 mL deionized water, 150 μL SDS (10%), 75 μL of 10% APS, and 15 μL TEMED. Following electrophoresis, the gels were fixed for 1 h in a 50:40:10 (*v*/*v*/*v*) water–ethanol–acetic acid solution, stained employing colloidal Coomassie Blue dye for 20 h, and followed by washing three times using dionized water. Protein bands were visualized and analyzed using the Chemidoc™ XRS+ Imaging System (Bio-Rad Laboratories, Hercules, CA, USA).

### 2.9. Microstructural Analysis Using Scanning Electron Microscopy

The microstructures of fermented CM and BM samples were assessed using scanning electron microscopy (SEM; JEOL JSM-6010LA, Akishima, Tokyo, Japan) at an accelerated voltage of 20 kV. Samples were lyophilized using a Telstar-cryodos freeze drier (Telstar, Terrassa, Spain), and small portions of the dried samples were mounted on aluminum SEM stubs using double-sided carbon tape before coating with a thin layer of gold using a Cressington 108 Auto Sputter Coater (Ted Pella Inc., Redding, CA, USA) [19]. Images were captured at 2000× and 3000× magnifications; however, only those taken at 3000× magnification were analyzed in this study.

### 2.10. Rheological Analysis

Dynamic oscillatory rheology was assessed in fermented CM and BM samples using a Discovery HR-2 hybrid rheometer (TA Instruments, New Castle, DE, USA) at 25 °C. The sample was gently mixed with a spoon, and a small amount was placed between 40 mm-diameter parallel plates with 100 µm gaps. Amplitude sweep tests were performed at 1 Hz to determine the linear viscoelastic region (LVE) of the samples within a range of 0.1–10%, founding 0.58% target strain as effective [36,37]. Frequency sweeps at 0.1–10 Hz were performed at this constant 0.58% strain to ensure that the applied deformation remained within the identified LVE range of the tested samples, to maintain linear viscoelastic response and structural integrity [37]. All measurements were performed in triplicate. The following rheological parameters were recorded using the TRIOS software (v2.3.1.1477, TA Instruments, New Castle, DE, USA): storage modulus (G′), loss modulus (G″), and tan delta (δ).

### 2.11. Statistical Analysis

All analytical tests and fermentations were performed in triplicate, and results are reported as means ± standard deviations (SDs). One-way ANOVA with Tukey’s post hoc test was used for mean comparisons, and two-way ANOVA was employed to analyze the effects of milk type and bacterial species on bacterial growth, TA%, pH, and OPA values, with *p* < 0.05 considered statistically significant. Pearson’s correlation analysis was used to examine the relationship between pH and TA% in fermented CM and BM samples. All statistical analyses were conducted using SPSS (IBM, Armonk, NY, USA, SPSS Statistics 29.0.0.0).

## 3. Results and Discussion

### 3.1. Bacterial Growth

**Figure 1:**
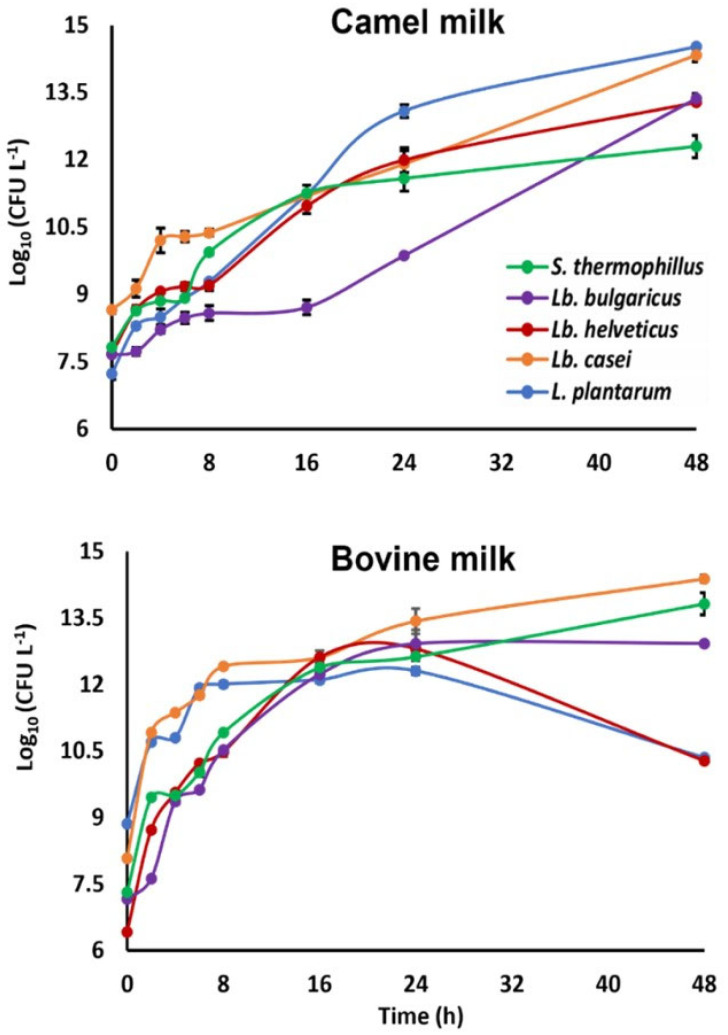
Growth of *Streptococcus thermophilus, Lactobacillus delbrueckii* ssp. *bulgaricus*, *Lactobacillus helveticus*, *Lactobacillus casei,* and *Lactiplantibacillus plantarum* in camel and bovine milk during 48 h fermentation at 42 °C. Values are means ± SDs (*n* = 3).

The growth of the commonly used traditional yogurt starter cultures *S. thermophilus* and *Lb. bulgaricus* as well as three alternative lactic acid bacteria, namely, *Lb. helveticus*, *Lb. casei,* and *L. plantarum*, in CM and BM fermented at 42 °C for 48 h was assessed along with their effects on the milk samples’ physicochemical characteristics. We acknowledge that the use of single strains does not accurately represent potential interactions between various bacteria, which are crucial in the actual product development [15,16,38]. However, this simple comparison was chosen to understand the unique behavior of each bacterium. All five strains exhibited faster growth in BM during the first 8 h, suggesting quicker early proliferation in these milk samples (Figure 1). This supports previous findings showing that BM facilitates rapid bacterial growth but is less effective in supporting long-term survival [39]. Notably, *Lb. bulgaricus* grew less efficiently in CM than in BM, whereas CM better supported the long-term growth of *Lb. helveticus*, *Lb. casei*, and *Lb. plantarum* than BM. Two-way ANOVA (Table 1) revealed that milk type and bacterial species significantly affected bacterial counts (*p* < 0.001), with no significant interaction effect, aligning with previous findings [7].

**Figure 2:**
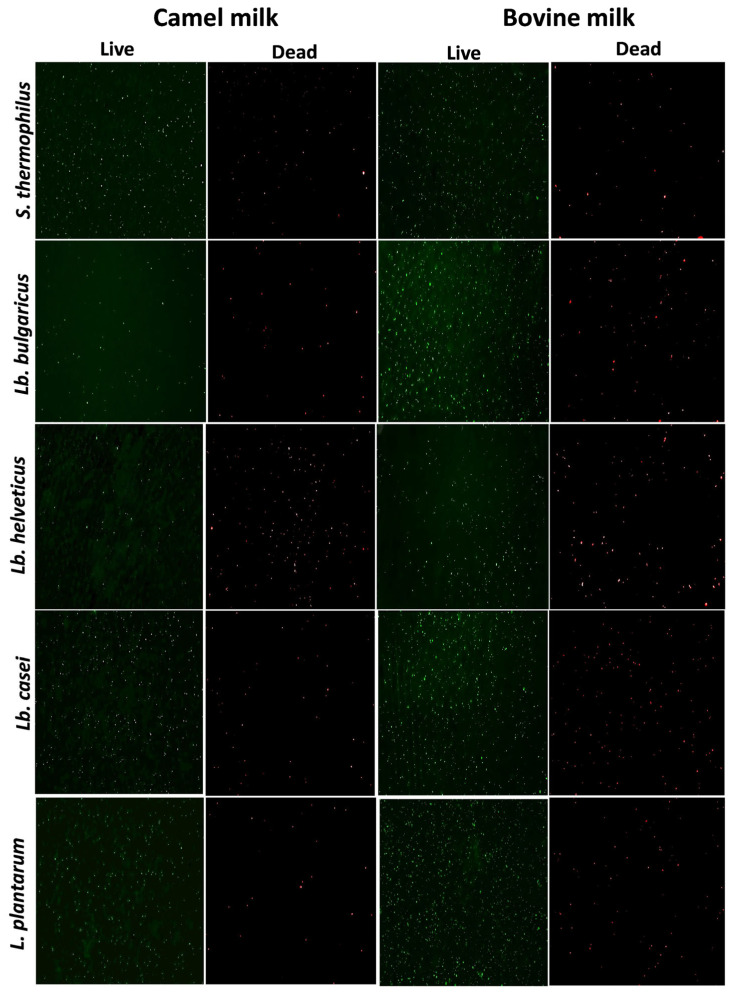
Live and dead bacteria analysis showing qualitative comparison between camel and bovine milk fermented with *Streptococcus thermophilus*, *Lactobacillus delbrueckii* ssp. *bulgaricus*, *Lactobacillus helveticus*, *Lactobacillus casei*, and *Lactiplantibacillus plantarum*. Green: live bacteria; red: dead bacteria.

Fluorescence microscopy revealed clear differences in bacterial viability between fermented CM and BM after 48 h of fermentation (Figure 2). This assessment was used mainly to observe bacterial cell integrity and membrane degradation during fermentation. The bacterial viability analysis in the fermented samples agrees with the viable counts observed in the bacterial growth curve. Bacterial survival was generally higher in BM than in CM. Notably, CM contains natural antimicrobial components, such as lysozymes, lactoferrins [4], and immunoglobulins [1], as well as a higher content of antimicrobial peptides [40], which may contribute to reduced bacterial viability.

### 3.2. TA and pH

**Figure 3:**
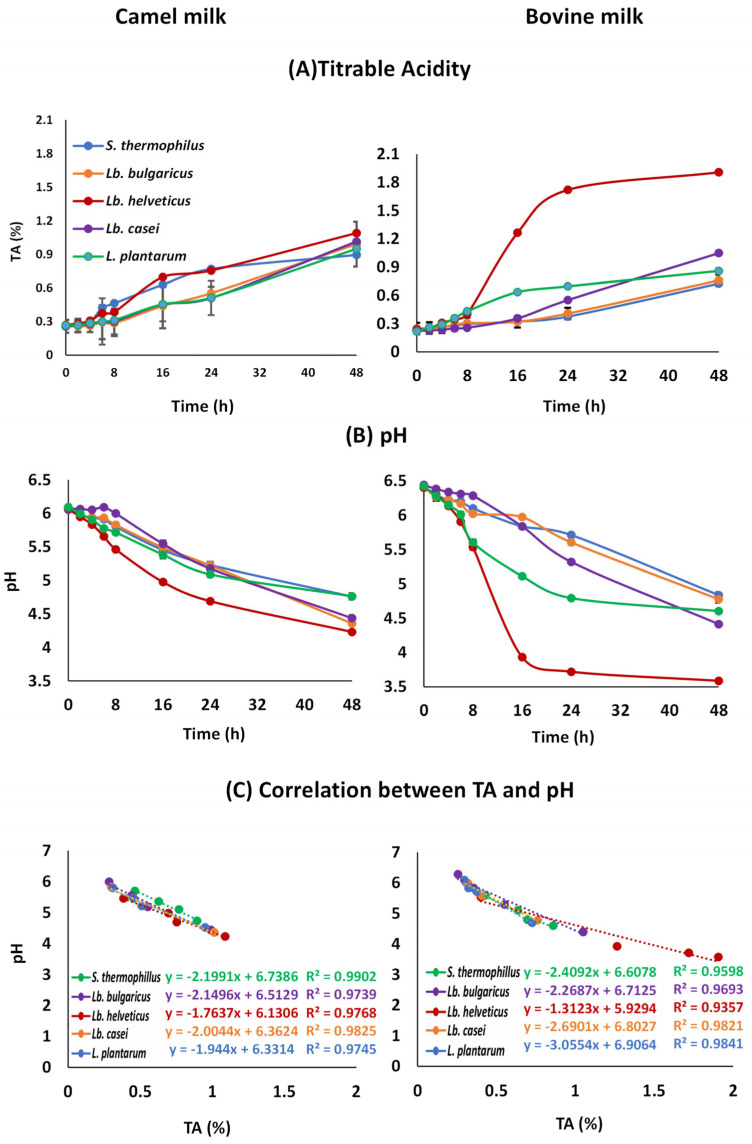
(**A**) Titratable acidity (TA%), (**B**) pH changes, and (**C**) correlation between TA% and pH in camel and bovine milk fermented with *Streptococcus thermophilus*, *Lactobacillus delbrueckii* ssp. *bulgaricus*, *Lactobacillus helveticus*, *Lactobacillus casei*, and *Lactiplantibacillus plantarum*. Values are means ± SDs (*n* = 3). TA%: No significant effect by milk type (M) (*p* > 0.05), significant effect by bacteria type (B) and their interaction (B × M) (*p* < 0.05); pH: No significant effect by milk type (M) (*p* > 0.05) and their interaction (B × M); significant effect by bacteria type (B) (*p* < 0.05).

Figure 3 illustrates changes in TA% and pH during the fermentation of CM and BM. Notably, among the five bacteria, *Lb. helveticus* showed the highest acidification, particularly in BM, i.e., higher TA% values and lower pH (*p* < 0.05). Except for *Lb. helveticus*, the decline in pH relative to TA% was lower in CM than in BM, suggesting CM’s stronger buffering capacity, consistent with [41]. Two-way ANOVA (Table 1) revealed significant effects of bacterial species (*p* < 0.001) and its interaction with milk type (*p* < 0.05) on TA% and pH, aligning with [7]. However, further research is required to clarify the underlying mechanisms. The difference in TA-pH correlation between the two milks is most likely owing to CM’s stronger buffering capacity due to its intrinsic properties, such as protein and phosphate content, which results in better pH drop resistance than BM [29].

### 3.3. Proteolysis During Fermentation

**Figure 4:**
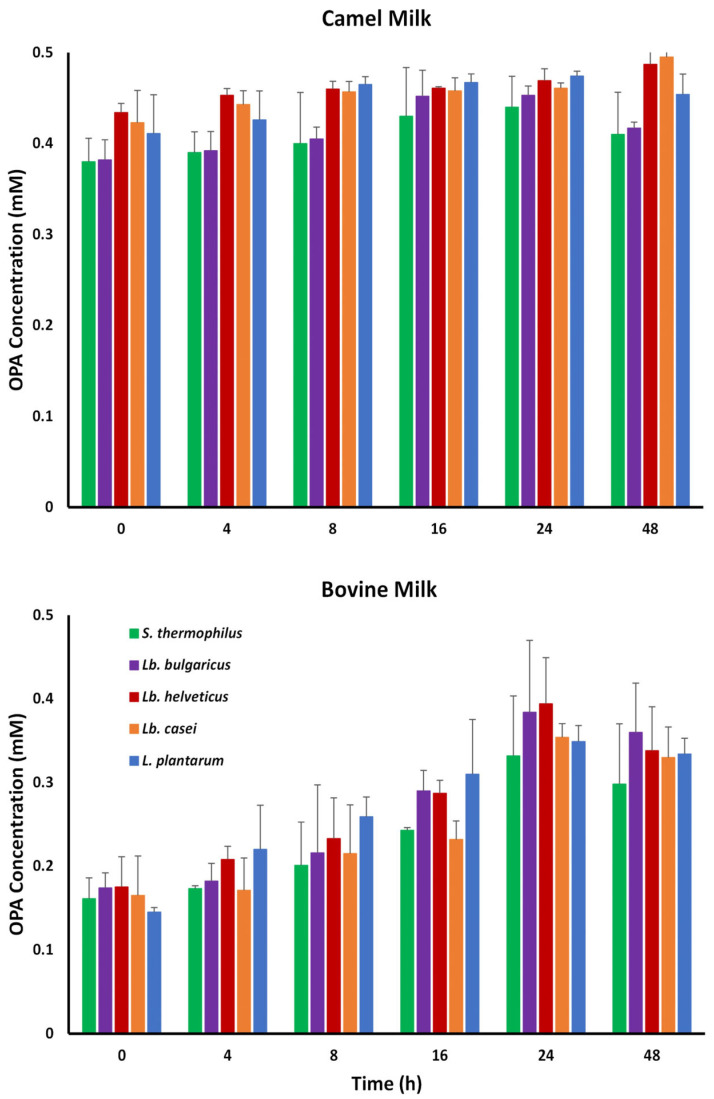
Degree of proteolysis (%) in camel and bovine milk fermented with *Streptococcus thermophilus, Lactobacillus delbrueckii* ssp. *bulgaricus*, *Lactobacillus helveticus*, *Lactobacillus casei,* and *Lactiplantibacillus plantarum*, determined using the ortho-phthalaldehyde (OPA) assay. Values are means ± SDs (*n* = 3). No significant effect by bacteria type (B) (*p* > 0.05); significant effect by milk type (M) and their interaction (B × M) (*p* < 0.05).

As shown in Figure 4, both unfermented and fermented CM samples exhibited consistently higher degrees of proteolysis than BM samples. Two-way ANOVA (Table 1) confirmed that proteolysis was significantly affected by milk type (*p* < 0.001) and by the interaction between milk type and bacterial species (*p* < 0.05), whereas bacterial strain alone did not show any significant effect. Notably, the OPA test assesses the overall quantity of free amino groups produced during protein breakdown and makes no distinction between different protease activities. Therefore, the observed variations represent differences in total proteolysis in the samples rather than strain-specific protease activities. Fermentation time had minimal impact on proteolysis in CM but led to a gradual increase in proteolysis in BM. Unfermented CM displayed stronger initial proteolysis and greater free amino acid content than unfermented BM, likely due to its enhanced plasmin/plasminogen activity [42]. These findings align with those of previous studies reporting increased free amino groups during BM fermentation [43]. SDS-PAGE (Figure S1) revealed significant differences in protein band profiles between unfermented and 48 h-fermented samples, supporting the OPA assay results indicating more extensive proteolysis in CM than in BM. This indicates the generation of bioactive peptides, including ACE-inhibitory sequences [44,45]; however, it may also increase bitterness by releasing hydrophobic casein-derived peptides [46]. Therefore, further peptide-level and sensory investigations are needed to understand the functional and sensory effects of CM proteolysis.

### 3.4. Microstructural Properties

**Figure 5:**
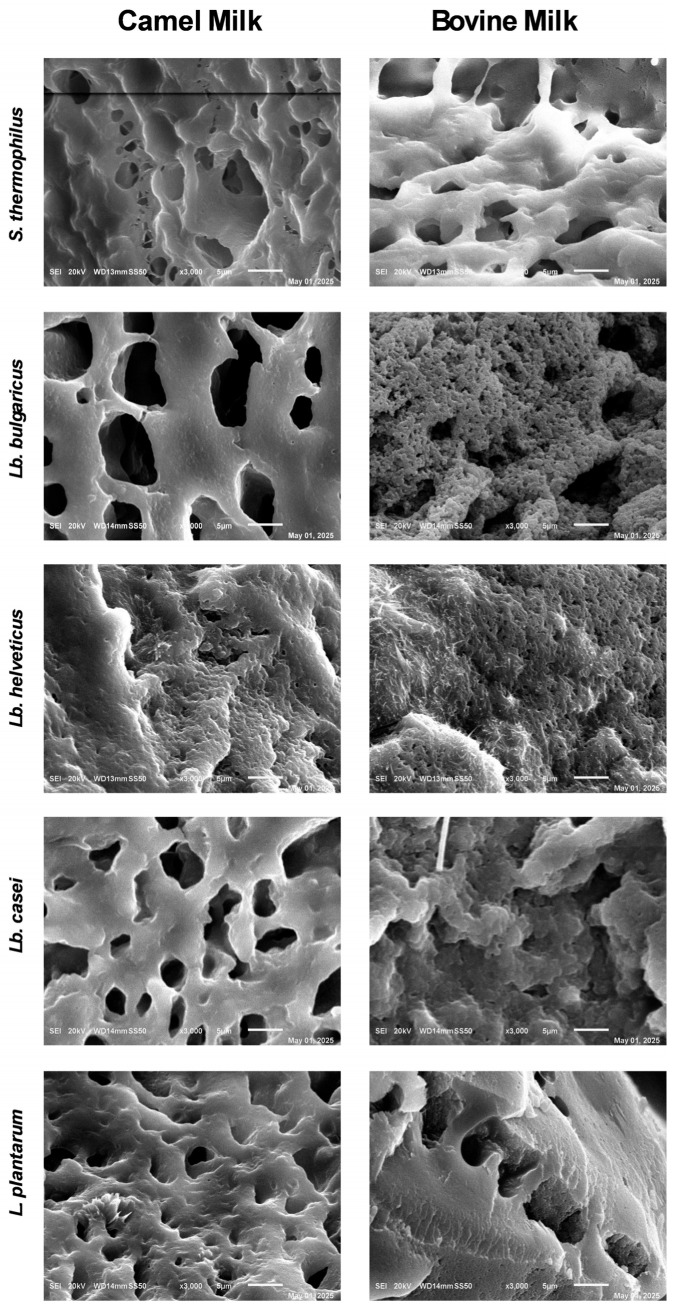
Scanning electron micrographs (3000×) of camel and bovine milk samples fermented with *Streptococcus thermophilus*, *Lactobacillus delbrueckii* ssp. *bulgaricus*, *Lactobacillus helveticus*, *Lactobacillus casei,* and *Lactiplantibacillus plantarum*.

Figure 5 presents SEM images illustrating distinct microstructural features in CM and BM fermented with the five lactic acid bacterial species. Across all bacterial treatments, fermented CM samples consistently showed smoother, more porous, and open gel networks, supporting a looser protein matrix compared with that of BM. Samples of CM and BM fermented with *Lb. helveticus*, *Lb. bulgaricus*, and *Lb. casei* exhibited smaller, less uniform pores and more compact structures than those fermented with *S. thermophilus* and *L. plantarum*. Among all samples, BM fermented with *Lb. bulgaricus* and *Lb. helveticus* appeared the most compact, whereas CM fermented with *L. plantarum*, *Lb. bulgaricus*, and *Lb. casei* showed the highest porosity. *Lb. helveticus* provided the densest fermented products from both CM and BM.

During fermentation, milk transitions from a low-viscosity Newtonian fluid to a semisolid or solid gel state [47]. Acidification markedly alters milk protein structure by promoting the formation of porous protein networks capable of entrapping serum [48]. Compared with fermented CM, BM formed denser protein networks upon fermentation [49,50]. The lower firmness of the CM coagulum is primarily attributed partly to the absence of β-lactoglobulin and the lower κ-casein content [51]. Bacterial fermentation lowers the pH of milk to the casein’s isoelectric point (CM 4.3 vs. BM 4.6), destabilizing micelles and promoting gel formation, whereas slower acidification and higher acidity in CM result in delayed gel firming [52,53]. SEM micrographs confirmed that fermented CM displayed more open microstructures, with thicker protein strands and wide, irregular pores, features associated with weak gels and reduced structural integrity, in agreement with previous reports [54,55]. In contrast, fermented BM showed denser casein networks with finer strands and smaller, more uniform pores, which may be correlated with its lower pH, contributing to a firmer texture and enhanced water-holding capacity [56]. Storage modulus G′ is significantly correlated with SEM-observed pore size and connectivity, as gels with smaller, more interconnected pores have larger storage moduli. Network structure and elasticity are associated by power-law [57] and fractal scaling models [58] in colloidal/protein aggregation gels. According to these models, the denser and robust microstructure of BM results in stronger gels with higher G′ and increased cross-linked density, opposed to the more open CM network [57,58]. Notably, the final pH differed between CM and BM, with CM typically reaching a slightly higher pH compared to BM, which further contributes to its fragile gel structure, as commercial starter cultures acidify CM at a slower rate compared with BM [59]. Overall, these microstructural differences play a critical role in shaping the rheological properties of the final product, which are influenced jointly by the milk composition and final pH and specific bacterial cultures employed during fermentation [11,12].

### 3.5. Rheological Properties

**Figure 6:**
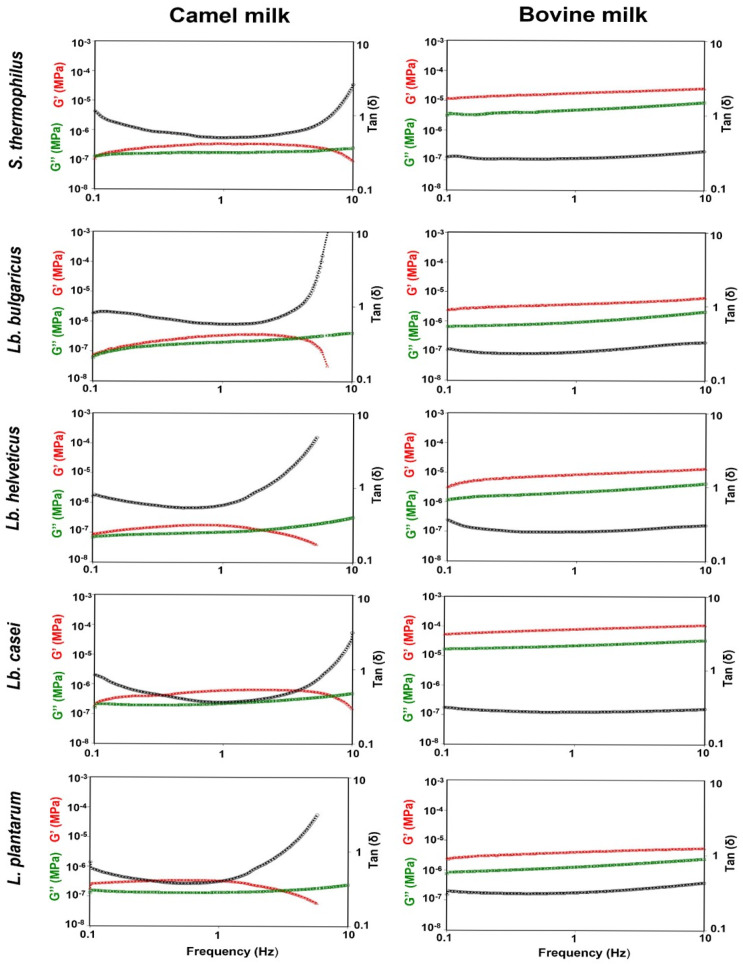
Rheological properties of camel and bovine milk fermented with *Streptococcus thermophilus*, *Lactobacillus delbrueckii* ssp. *bulgaricus*, *Lactobacillus helveticus*, *Lactobacillus casei,* and *Lactiplantibacillus plantarum*. The storage modulus (G′) is shown in red, the loss modulus (G″) is shown in green, and the tan (δ) is shown in black.

Rheological analysis revealed significant differences between fermented CM and BM for all bacterial strains. CM consistently exhibited significantly lower storage modulus (G′, indicating elastic solid gel-like behavior) relative to loss modulus (G″, indicating viscous fluid-like behavior). In contrast, BM had higher and more variable G′/G″ ratios (*p* < 0.001), as reflected by higher tan δ values for CM (Figure 6). These findings were consistent with SEM observations, which showed different gel structures and more fluid-like behavior in fermented CM and a stronger gel-like structure in fermented BM [20]. All five bacteria yielded stable, low tan δ values in fermented BM, confirming stronger, more elastic gels. In contrast, CM gels were loose, open, porous, and weak, possibly due to the accumulation of water [54]. According to [49], the pH, acidification kinetics, heating temperatures and duration, and protein types and concentrations all might affect the microstructural and rheological attributes of acid gel. Notably, fermented CM products had higher final pH values than BM gels. Reducing the pH of milk to a level closer to the casein isoelectric point (~4.6 for BM, ~4.3 for CM) accelerates the gelation process and increases the storage modulus (G′), producing a stronger and more cohesive protein network [60]. Spray-dried reconstituted skimmed milk powder can denature whey proteins and change mineral distribution, potentially impacting fermentation and texture [61]; this is acknowledged as a restriction, with future research requiring raw whole milk.

## 4. Conclusions

This study provided a comparative evaluation of bacterial growth, acidification, proteolysis, and rheological properties in CM and BM fermented with five lactic acid bacteria: *S. thermophilus*, *Lb. delbrueckii* ssp. *bulgaricus*, *Lb. helveticus*, *Lb. casei*, and *L. plantarum*. Overall, BM supported faster bacterial growth, more rapid acidification, and stronger gel formation than CM. These differences are largely attributed to compositional factors, including the absence of β-lactoglobulin, low level of κ-casein, high and slow acidification, and high proteolysis rate in CM, which contribute to its weak gel structure and watery consistency. SEM revealed that fermented CM forms a looser protein matrix with larger pores and lower density compared with fermented BM. These microstructural differences were consistent with rheological findings, where CM exhibited higher tan δ values than BM. Compared with the other bacteria, growth patterns, metabolic properties, mild acidity, proteolytic activity, and long-term survival of *Lb. casei* in CM highlights its potential suitability for developing probiotic or functional dairy products. Sensory analysis and long-term stability evaluation (during storage conditions) were not performed in this study; however, a thorough assessment of sensory and general acceptability and shelf-life stability of fermented CM and BM yogurt needs to be explored. Moreover, future research may focus on increasing CM gel strength, e.g., by employing techniques such as enzymatic cross-linking, the addition of hydrocolloids, or the use of mixed cultures. Additionally, studies assessing consumer preferences, beyond drinkable fermented CM (laban) vs. CM yogurt, and the economic viability of using *Lb. casei* are warranted. In addition, accepting the textural and rheological limitations of CM and developing special drinkable fermented CM products is a viable alternative.

## Figures and Tables

**Table 1:** *p*-values from two-way ANOVA assessing the influence of bacterial strain and milk type on microbial growth, acidification, and proteolysis.

Dependent Variables	Independent Variables and Interactions		
	Bacteria (B)	Milk Type (M)	B × M
**Bacterial count**	0.001 *	0.001 *	NS
**TA (%)**	0.001 *	NS	<0.05 *
**pH**	0.001 *	NS	NS
**OPA**	NS	0.001 *	<0.05 *

Abbreviations: TA (titratable acidity), OPA (*ortho*-phthalaldehyde assay); values marked with an asterisk (*) indicate statistically significant effects (*p* < 0.05); NS (not significant) (*p* > 0.05).

## Data Availability

The original contributions presented in this study are included in the article/Supplementary Material. Further inquiries can be directed to the corresponding author.

## Supplementary Materials

The following supporting information can be downloaded at: https://www.mdpi.com/article/10.3390/foods15030546/s1. Figure S1: SDS-PAGE profiles of unfermented and fermented camel and bovine milk treated with *Streptococcus thermophilus*, *Lactobacillus delbrueckii* ssp. *bulgaricus*, *Lactobacillus helveticus*, *Lactobacillus casei*, and *Lactiplantibacillus plantarum*.

## Abbreviations

CM	Camel Milk
BM	Bovine Milk
SEM	Scanning Electron Microscopy
