# Supplementary material for: Microstructural and Rheological Properties of Camel and Bovine Milk Fermented with Five Lactic Acid Bacteria Strains
Source: Foods. 2026 Feb 4;15(3):546. doi: 10.3390/foods15030546 (PMC12896414; doi:10.3390/foods15030546)
Supplement: Supplementary file 1 [file foods-15-00546-s001.zip › foods-4113037-supplementary.pdf]

Supplementary Materials:

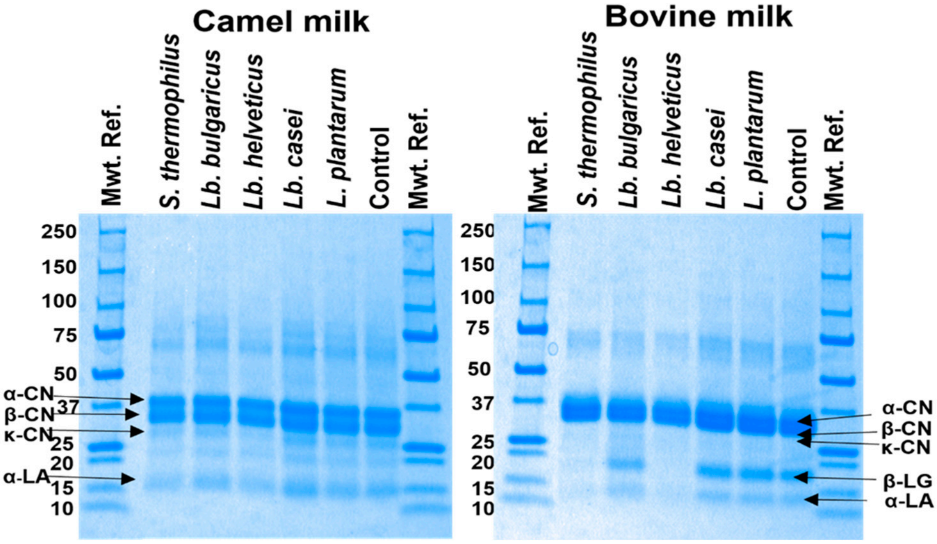

Figure S1. SDS-PAGE profiles of unfermented and fermented camel and bovine milk treated with Streptococcus thermophilus, Lactobacillus delbrueckii ssp. bulgaricus, Lactobacillus helveticus, Lactobacillus casei, and Lactiplantibacillus plantarum. Abbreviations: CN, casein; LG, lactoglobuin; LA, lactoalbumin.
